# Reconstruction of Single-Cell Spatial Transcriptomes in Archival Kidney Biopsies

**DOI:** 10.1016/j.ekir.2025.11.036

**Published:** 2025-12-05

**Authors:** Simen F. Pettersen, Eleni Skandalou, Jessica Furriol, Tarig Osman, Hans Peter Marti, Øystein Eikrem

**Affiliations:** 1 Department of Clinical Medicine, University of Bergen, Bergen, Norway; 2 Medical Department, Haukeland University Hospital, Bergen, Norway

**Keywords:** spatial transcriptomics, Visium HD, FFPE, podocyte

## Introduction

The postgenomic era brought major advancements in biological research, culminating in single-cell RNA sequencing in the late 2010s. However, single-cell RNA sequencing requires dissociation of tissues, thus losing spatial context. Recognizing the importance of spatial cell relationships, *Nature* named spatially resolved transcriptomics its 2020 Method of the Year.^1^ At that time, single cell resolution in spatial transcriptomics was not feasible, but emerging tools such as Visium HD (10x Genomics, Pleasanton, CA) now achieve 2 × 2 μm (4 μm^2^) spatial probe-based transcriptomics.^2^ Visium HD data are typically aggregated across 16 adjacent probe spots to approximate a “typical” human cell area (8 x 8 μm = 64 μm^2^), increasing signal but risking mixed transcripts form heterogenous cell populations. However, with its subcellular resolution, Visium HD enables reconstruction of single-cell transcriptomes through deep learning–based nuclear segmentation in the hematoxylin and eosin image. To this end, StarDist^3^ has proven to be effective.

Kidney biopsies involve extraction of core needle samples (1.2 x 20 mm), which are then processed as formalin-fixed, paraffin-embedded (FFPE) tissue for subsequent evaluation. Following diagnostics, FFPE blocks are archived in room temperature for years. Spatial transcriptomic analysis of FFPE material expands their translational value and has become a key approach in kidney research. Highlighting this, a recent review by Isnard and Humphreys identified 37 studies spanning kidney development, healthy tissue, and chronic kidney disease.^4^ The kidney’s distinct morphology and disease-related structures, such as the glomerulus in nephrotic syndrome, will further drive spatial transcriptomics in this field. FFPE tissues therefore represent an important resource. Building on our previous demonstration of bulk RNA sequencing feasibility in FFPE kidney biopsies,^5^ we now extend this to reconstruct single-nucleus spatial transcriptomic data from archival samples of patients with idiopathic nephrotic syndrome. A full protocol is provided in the Supplementary Methods.

## Results

The patients’ clinical characteristics are summarized in Supplementary Table S2. In total, 9552 genes (counts ≥ 3) were detected across 5058 nuclei from FFPE biopsies stored for 20, 20, and 11 years. Despite recommendations to use samples stored for < 5 years, our findings below demonstrate that older biopsies can still yield valuable and translationally relevant data.

**Figure 1:**
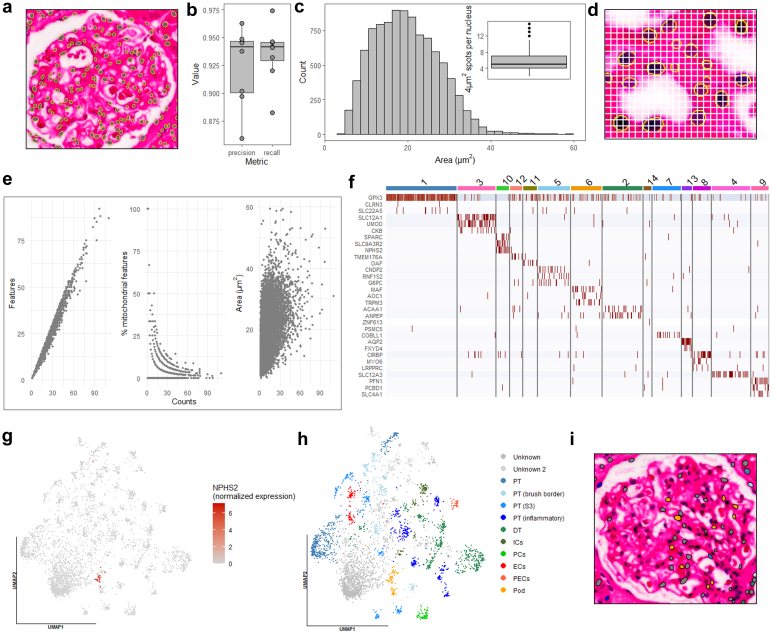
Analysis of steroid-sensitive nephrotic syndrome patient dataset. (a) StarDist nuclei segmentation of glomerular region of interest. (b) StarDist segmentation performance for all glomerular regions of interest evaluated by precision and recall. (c) Histogram showing the area distribution of segmented nuclei. Inset shows the number of 4 μm^2^ spots binned together within each nucleus. (d) Related to (c), zoomed in view of segmented nuclei with a grid overlay of 4 μm^2^ spots. (e) Scatter plots showing how the number of features, % mitochondrial features, and nuclear area are related to the unique molecular identifier counts. (f) Heatmap showing the scaled expression of the top 3 markers in each cluster. (g) Uniform manifold approximation and projection embedding of nuclei showing expression of canonical podocyte marker gene *NPHS2*. (h) The same uniform manifold approximation and projection embedding as in (f) showing cell type or state assignment. (i) Segmented nuclei in glomerular region of interest with cell type or state assignment. Note the reduction in number of nuclei because of filtering based on total RNA counts and % mitochondrial genes. DT, distal tubule; ECs, endothelial cells; ICs, intercalated cells; PCs, principal cells; PECs, parietal epithelial cells; Pod, podocyte; PT, proximal tubule.

We first analyzed each patient dataset individually (Supplementary Table S1), presenting results from the steroid-sensitive nephrotic syndrome case. The StarDist model faithfully segmented nuclei, achieving mean precision and recall ˃ 90% within glomeruli (Figure 1a and b). For each segmented nucleus, 4 μm^2^ capture areas were binned together and their transcription profiles summated. The area distribution of the segmented nuclei centered around 20 μm^2^, averaging five 4 μm^2^ spots per nucleus (Figure 1c and d). We found that nuclei area was moderately positively associated with counts per nucleus (R^2^ = 0.21, *P* < 2.2 × 10^−16^) (Figure 1e). To obtain a high-quality dataset for downstream analyses, filtering on the number of counts per nucleus (> 10) and percentage of mitochondrial genes per nucleus (< 5%) was applied, with the former having the greatest impact (Supplementary Figure S1A and B). The filtered dataset consisted of 3994 nuclei with mean counts of 21.08 ± 10.72 and mean features of 19.52 ± 9.69 (Supplementary Figure S1C). Next, the unsupervised clustering identified 15 clusters (Supplementary Figures S1D–G, Figure S2, and S3). Comparison of count and feature distributions across clusters revealed that most pairwise comparisons showed negligible to small differences (|Hedges’ g| < 0.5), whereas a subset displayed moderate to strong differences (|Hedges’ g| ≥ 0.5). These differences did not compromise cluster assignment confidence, as all clusters exhibited high median assignment scores ranging from 0.8 to 1.0 (Supplementary Figure S4). Intercluster differential expression analysis identified cluster marker genes (Figure 1f), enabling assignment of clusters into 12 cell types or states (Figure 1h). Podocytes were classified based on the expression of canonical podocyte marker gene, *NPHS2*^6^ (Figure 1g). Glomerular endothelial cell and mesangial cell origin of the podocyte cluster was dismissed because of lack of expression of glomerular endothelial cell or mesangial cell marker genes *PECAM1*, *PLVAP*, *EHD3*, *CLIC4*, and *PDGFRB*^7^^,^^8^ (Supplementary Figure S5). Spatial plotting confirmed its podocyte origin as it localized to glomeruli (Figure 1i and Supplementary Figure S6).

**Figure 2:**
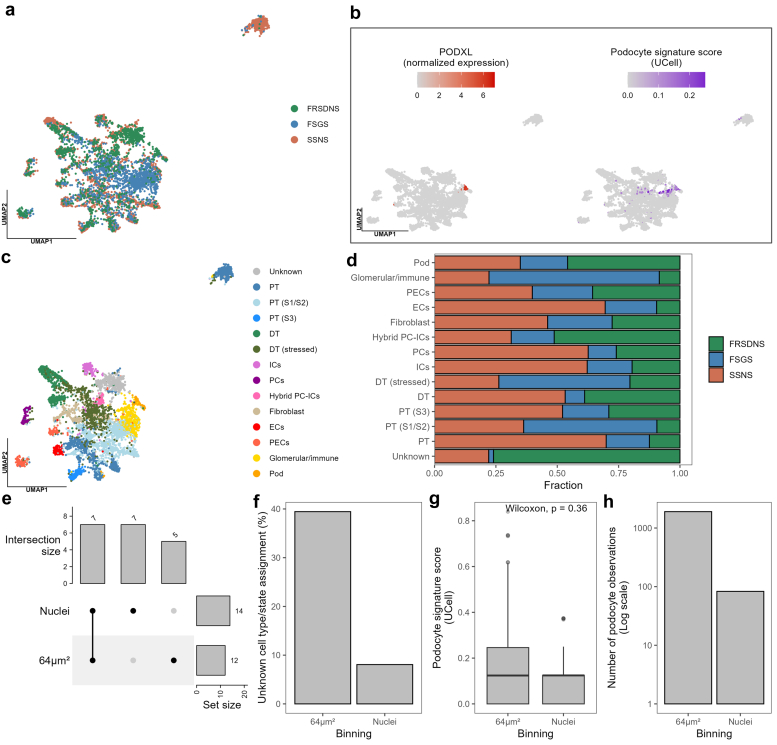
Analysis of integrated idiopathic nephrotic sydrome patient dataset. (a–d) Canonical correlation analysis was used to integrate single-nucleus spatial transcriptomic datasets from 3 patients with idiopathic nephrotic sydrome, and (e–h) the nuclei binning was compared to standard 64 μm^2^ binning. (a) UMAP embedding of nuclei showing patient origin. (b) UMAP embedding of nuclei showing expression of canonical podocyte marker gene *PODXL* and podocyte gene signature score computed with UCell. (c) UMAP embedding showing cell type or state assignment. (d) Stacked bar plot showing the cell type or state coverage within each patient. (e) Upset plot showing the number of shared and unique cell types or states identified with nuclei binning or 64 μm^2^ binning. (f) Bar plot showing the percentage of observations assigned to unknown cell type or state with nuclei binning or 64 μm^2^ binning. (g) Comparison of podocyte gene signature scores for the podocyte cluster identified with nuclei binning or 64 μm^2^ binning (Wilcoxon rank sum test, α = 0.05). (h) Bar plot showing the number of observations within the podocyte cluster identified with nuclei binning or 64μm^2^ binning. DT, distal tubule; ECs, endothelial cells; FRSDNS, frequently relapsing steroid-dependent nephrotic syndrome; FSGS, focal segmental glomerulosclerosis; ICs, intercalated cells; PCs, principal cells; PECs, parietal epithelial cells; Pod, podocyte; PT, proximal tubule; SSNS, steroid-sensitive nephrotic syndrome; UMAP, uniform manifold approximation and projection.

Next, we used canonical correlation analysis to integrate the 3 datasets (Figure 2a and Supplementary Figure S7). Importantly, the identified clusters represented cell types or states conserved across the different patients rather than technical batch effects. Cell type or state assignment was done by identifying cluster-specific marker genes and with manually curated gene signatures. Podocytes were identified based on the expression of *PODXL* and a ∼10-fold increase in podocyte gene signature score compared to other clusters (Figure 2b). In total, 14 cell types or states were detected in the integrated dataset (Figure 2c). Of note was the separation of 1 proximal tubule cluster that separated from other proximal tubules and derived mostly from the patient with steroid-sensitive nephrotic syndrome (Figure 2a and c). This separate cluster had *GPX3* as the main marker, whereas other proximal tubule clusters also had markers such as *DPEP1* and *ANPEP*. We interpret this as an artifact of transcript degradation in old biopsies, wherein the same cell type can be represented by different clusters depending on which transcripts are degraded or not. All cell types or states had coverage from the 3 clinical phenotypes, albeit at varying levels (Figure 2d and Supplementary Figure S8). Given a higher powered study design, such cell type or state distributions could potentially correlate to clinical phenotypes.

Lastly, we compared nuclei binning to standard 64 μm^2^ on the integrated datasets. Using the same filtering and clustering parameters, we identified fewer cell types or states with 64 μm^2^ binning (14 vs. 12) (Figure 2e and Supplementary Figure S9). Furthermore, the percentage of bins that could not confidently be assigned to a cell type or state was higher with 64 μm^2^ binning (39% vs. 8%) (Figure 2f). Focusing on the podocyte clusters identified for the 2 binning approaches, we found no statistically significant difference in podocyte gene signature scores (Wilcoxon *P* = 0.36) (Figure 2g). However, the 64 μm^2^ binning had an ∼10-fold increase in number of podocyte observations (Figure 2h).

## Discussion

These results indicate that the signal deconvolution achieved with nuclei binning is more effective for identification of niche cell types or states, such as the hybrid PC-IC population. This is further supported by the reduced proportion of ambiguous cell type or state assignments observed with nuclei binning. For more common cell types, such as podocytes, the smaller area—and thus signal—does not negatively impact gene signature detection. However, for applications such as differential expression analysis, 64 μm^2^ binning may be advantageous because increased statistical power. The caveat is that transcripts from different cell populations may produce mixed signals, especially in heterogenous tissue compartments, like glomeruli. A potential middle-ground approach would be to expand nuclear regions to approximate cell boundaries; however, this comes with the risk of losing true single-cell resolution, as cell boundaries are not reliably defined by consistent morphological features. Further discussions are presented in the Supplementary Text.

In conclusion, for archival FFPE kidney biopsies, single-cell level information can be reconstructed by leveraging the subcellular resolution of Visium HD combined with morphological cell features in the hematoxylin and eosin image. The data obtained through this approach is of sufficient quality to faithfully identify conserved cell types across patients, as well as for identifying niche cell types or states.

## Disclosure

All the authors declared no competing interests.

## Patient Consent

The authors declare that they have obtained written consent from the patients discussed in the report. This study was approved by the Regional Committee for Medical and Health Research Ethics (REC WEST) under approval number 2013/553.

## Supplementary Material

Supplementary File (PDF) **Supplementary Methods. Supplementary References. Supplementary Text. Figure S1.** snRNA-seq processing of SSNS dataset. **Figure S2.** snRNA-seq processing of FSGS dataset. **Figure S3.** snRNA-seq processing of FRSDNS dataset. **Figure S4.** Cluster quality assessment of SSNS dataset. **Figure S5.** Cluster marker genes for glomerular cells in SSNS dataset. **Figure S6.** Spatial visualization of podocytes in SSNS patient biopsy. **Figure S7.** Data integration using CCA. **Figure S8.** Over/underrepresentation of cell types/states for the 3 patients. **Figure S9.** UMAP embeddings of integrated dataset based on 8 x 8 μm binning. **Table S1.** Parameters used for unsupervised clustering. **Table S2.** Patients’ clinical characteristics. STROBE checklist.

## Footnotes

Supplementary File (PDF)

**Supplementary Methods.**

**Supplementary References.**

**Supplementary Text.**
**Figure S1.** snRNA-seq processing of SSNS dataset. **Figure S2.** snRNA-seq processing of FSGS dataset. **Figure S3.** snRNA-seq processing of FRSDNS dataset. **Figure S4.** Cluster quality assessment of SSNS dataset. **Figure S5.** Cluster marker genes for glomerular cells in SSNS dataset. **Figure S6.** Spatial visualization of podocytes in SSNS patient biopsy. **Figure S7.** Data integration using CCA. **Figure S8.** Over/underrepresentation of cell types/states for the 3 patients. **Figure S9.** UMAP embeddings of integrated dataset based on 8 x 8 μm binning. **Table S1.** Parameters used for unsupervised clustering. **Table S2.** Patients’ clinical characteristics. STROBE checklist.
