# Supplementary material for: Reconstruction of Single-Cell Spatial Transcriptomes in Archival Kidney Biopsies
Source: Kidney Int Rep. 2025 Dec 5;11(2):103713. doi: 10.1016/j.ekir.2025.11.036 (PMC12805023; doi:10.1016/j.ekir.2025.11.036)
Supplement: Supplementary File (PDF) — Supplementary Methods. Supplementary References. Supplementary Text. Figure S1. snRNA-seq processing of SSNS dataset. Figure S2. snRNA-seq processing of FSGS dataset. Figure S3. snRNA-seq processing of FRSDNS dataset. Figure S4. Cluster quality assessment of SSNS dataset. Figure S5. Cluster marker genes for glomerular cells in SSNS dataset. Figure S6. Spatial visualization of podocytes in SSNS patient biopsy. Figure S7. Data integration using CCA. Figure S8. Over/underrepresentation of cell types/states for the 3 patients. Figure S9. UMAP embeddings of integrated dataset based on 8 x 8 μm binning. Table S1. Parameters used for unsupervised clustering. Table S2. Patients’ clinical characteristics. STROBE checklist. [file mmc1.pdf]

Supplementary Methods

Sample preparation, library preparation and sequencing

The capture area in Visium HD platform is 6.5 x 6.5 mm. To use this area to its fullest potential, the kidney biopsies paraffin blocks were melted down and kidney cores coming from different biopsies were rearranged together in pairs or triplets in new paraffin blocks. The cores were placed parallel to each other in a way that they fit the capture area. After the samples' rearrangement, sections of 4 µm thickness were collected in a water bath with MilliQ water (42°C), floating on the water surface until flattening. The sections were placed on SuperFrost plus slides (VWR SuperFrost Plus, Adhesion Slides). Biopsy sections were oriented parallel to the top and bottom edges of the slides, ensuring that all tissue areas were positioned at least 15 mm from the top and bottom edges and 5 mm from the sides. Slides were subsequently dried for 3 hours at 42 °C and stored at room temperature in a desiccator, following the guidelines outlined in the *Visium HD FFPE Tissue Preparation Handbook* [S1].

Hematoxylin and Eosin staining was performed according to the 10x Genomics *Visium HD FFPE Tissue Preparation Handbook* [S1]. Library preparation followed the 10x Genomics *Visium HD Spatial Gene Expression Reagent Kits* [S2], and sequencing was carried out according to the *Element AVITI System User Guide Chapter 5* [S3], using the PN 860-00015 AVITI 2x75 Sequencing Kit Cloudbreak FS High Output. Staining, library preparation and sequencing were performed at the Genomics Core Facility, Oslo University Hospital. Imaging was conducted using the Olympus VS200 Slide Scanner at 40X magnification. Spatial sequencing raw data was processed using Space Ranger (version 3.1.1) from 10x Genomics.

Nuclei segmentation and custom binning

Python (version 3.13) was used for nuclei segmentation and custom binning. The Space Ranger outputs for the 2x2 µm capture areas were loaded into Python as anndata objects from scverse [S4]. Segmentation of nuclei in the H&E image and binning of capture areas was performed using the method described in the analysis guide from 10x Genomics [S5]. Briefly, the high resolution H&E input image was percentile normalized using 5% and 95% as the minimum and maximum percentiles, respectively. For nuclei segmentation we used the pretrained 2D versatile H&E model from StarDist [3], with "block_size" = 4096, "prob_thresh" = 0.2, "nms_thresh" = 0.05, "min_overlap" = 128 and "n_tiles" = (10, 1, 1). The segmentation results and the tissue positions of the 2x2 µm capture areas were converted into two separate Geodataframes using GeoPandas (version 1.0.1). The two Geodataframes were then combined, keeping only the spatial barcodes localized to segmented nuclei. The identified barcodes were filtered to only retain barcodes uniquely assigned to one nucleus, and this subset of barcodes was used to filter the anndata object. Next, gene-wise summation of unique molecular identifier (UMI) counts from barcodes localizing to the same nucleus was applied to the anndata object. Lastly, the anndata object was filtered to only keep nuclei with an area < 60 μm^2.

snRNA-seq processing

The snRNA-seq data from the nuclei segmentation and custom binning were processed in RStudio (version 2024.12.1) using the Seurat package (version 5.2.1) [S6]. The datasets for each patient were first analyzed individually using the following method: First, the anndata object was imported into RStudio

as a Seurat object using the h5ad2seurat() function from the schard package (version 0.0.1). All Seurat objects were filtered to exclude nuclei with a percentage of mitochondrial features ≥ 5%. The Seurat objects were also filtered based on number of counts, using a count threshold that gave a good unsupervised clustering result without excluding too many nuclei. The count thresholds were > 10, >15, and > 15 for the SSNS, FRSDNS, and FSGS cases, respectively. Unsupervised clustering was performed using the standard Seurat workflow: 1) The data was normalized, 2) the top 2000 variable features were identified, 3) the data was scaled based on the identified variable features, 4) principal component analysis (PCA) was used to determine the dimensionality of the data, 5) nuclei were embedded in a K-nearest neighbors (KNN) graph based on the identified PCA dimensions, 6) the Louvain algorithm was used to identify clusters of cells from the KNN graph, 7) UMAP embedding was applied for visualization of identified clusters. Table S1 summarizes the PCA components- and clustering resolution used for each of the three datasets. Marker genes were identified using the FindAllMarkers() function. Prior to integration, all Seurat objects were subset to exclude cluster 0 since it did not have any identified markers in any of the three patient datasets.

Table S1: Parameters used for unsupervised clustering.

Dataset	PCA components	Clustering resolution
SSNS	1:15	0.2
FRSDNS	1:10	0.2
FSGS	1:10	0.2

For CCA integration, the trimmed Seurat objects were used to select integration features using the SelectIntegrationFeatures() function. These integration features were then used as input to the FindIntegrationAnchors() function, and the identified anchor set was then used as input to the IntegrateData() function. The integrated dataset was then clustered using the same unsupervised clustering workflow as outlined above (with PCA components 1:20 and clustering resolution = 1). Inter-patient conserved cluster markers were identified using the FindConservedMarkers() function. Podocyte gene signature scores were calculated with UCell [S7] using the following geneset: *PODXL*, *NPHS1*, *NPHS2*, *CLIC5*, *SPOCK2*, *BCAM*, *SLC9A3R2*, *PLA2R1*. Plots were produced using built in functions in Seurat and ggplot2 (version 3.5.1) [S8].

8x8 µm binned datasets were processed in the same manner as their respective nuclei binned datasets, as well as the integration.

Supplementary Results

Table S2 Patients' clinical characteristics

	All patients (n=3)
Sex (male/female)	3/0
Age (median; range)	23 (17-27)
eGFR, mL/min/1.73m^2 (median; range)	127 (44-131)
Serum albumin, g/L (median; range)	31 (20-41)
Proteinuria, dipstick crosses (1+/2+/3+/4+)	0/0/2/1
Years post biopsy (median; range)	20 (11-20)

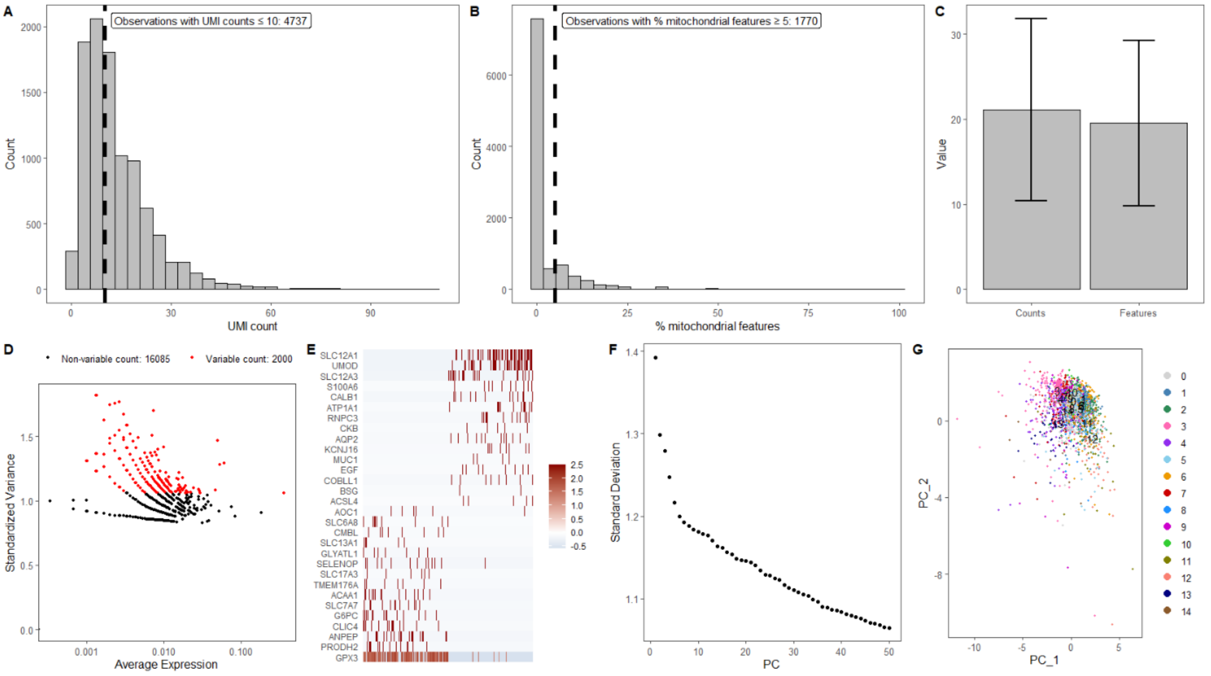

Figure S1: snRNA-seq processing of SSNS dataset. a) Histogram of UMI counts. The dashed vertical line indicates the lower threshold. **b)** Histogram of % mitochondrial features. The dashed vertical line indicates the upper threshold. **c)** Mean UMI counts and features ± SD after filtering. **d)** Identification of top 2000 variable features used for scaling the dataset. **e)** Heatmap showing the genes and scaled expression for the top 15 loading scores in the positive and negative direction along the first principal component. **f)** Elbow plot showing the reduction in the variation as a function of the number of principal components. **g)** PCA plot of the identified clusters along the first two principal components.

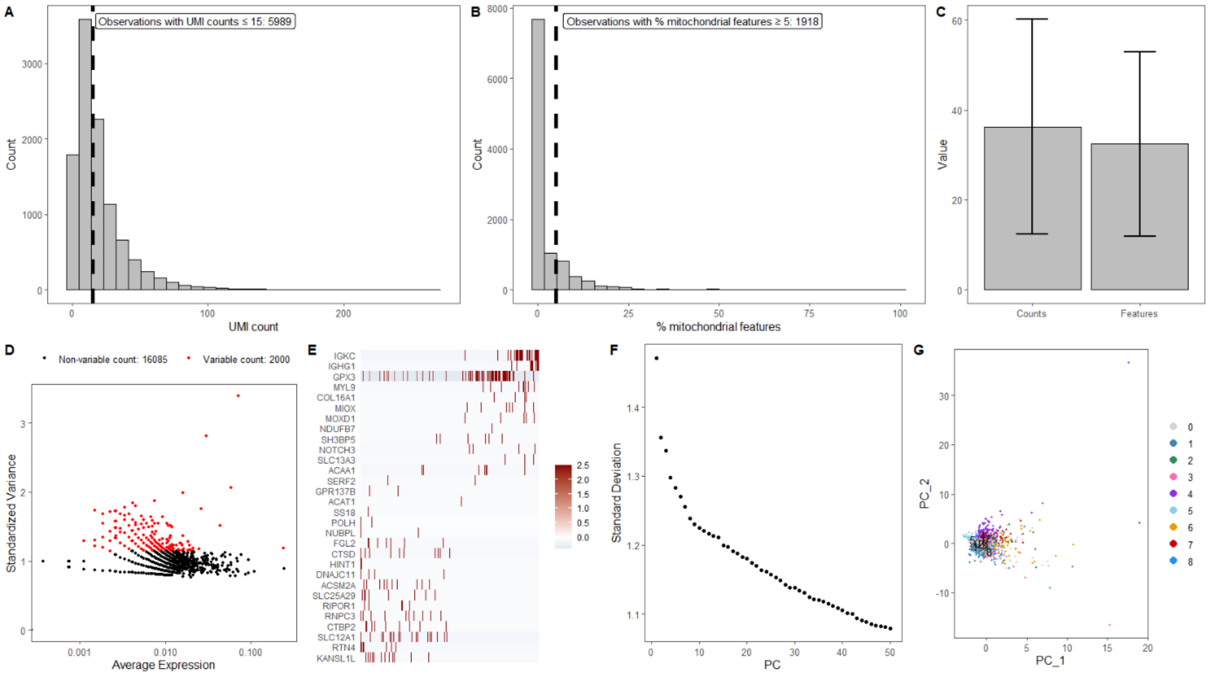

Figure S2: snRNA-seq processing of FSGS dataset. a) Histogram of UMI counts. The dashed vertical line indicates the lower threshold. **b)** Histogram of % mitochondrial features. The dashed vertical line indicates the upper threshold. **c)** Mean UMI counts and features ± SD after filtering. **d)** Identification of top 2000 variable features used for scaling the dataset. **e)** Heatmap showing the genes and scaled expression for the top 15 loading scores in the positive and negative direction along the first principal component. **f)** Elbow plot showing the reduction in the variation as a function of the number of principal components. **g)** PCA plot of the identified clusters along the first two principal components.

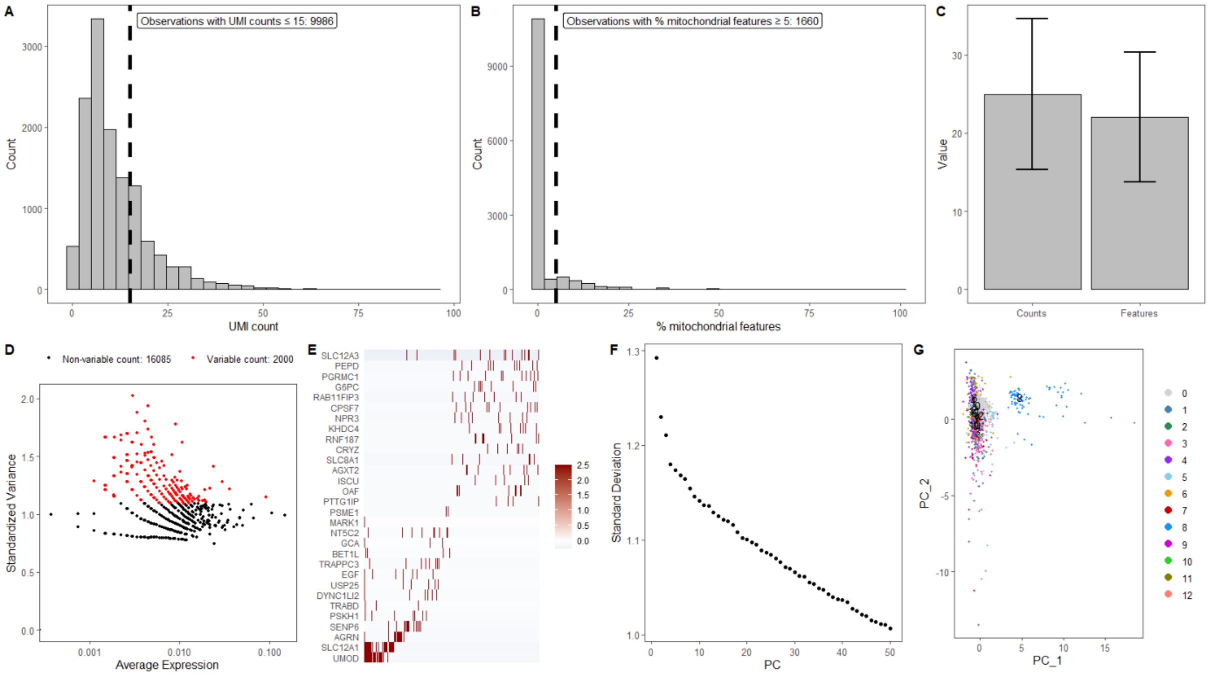

Figure S3: snRNA-seq processing of FRSDNS dataset. a) Histogram of UMI counts. The dashed vertical line indicates the lower threshold. **b)** Histogram of % mitochondrial features. The dashed vertical line indicates the upper threshold. **c)** Mean UMI counts and features ± SD after filtering. **d)** Identification of top 2000 variable features used for scaling the dataset. **e)** Heatmap showing the genes and scaled expression for the top 15 loading scores in the positive and negative direction along the first principal component. **f)** Elbow plot showing the reduction in the variation as a function of the number of principal components. **g)** PCA plot of the identified clusters along the first two principal components.

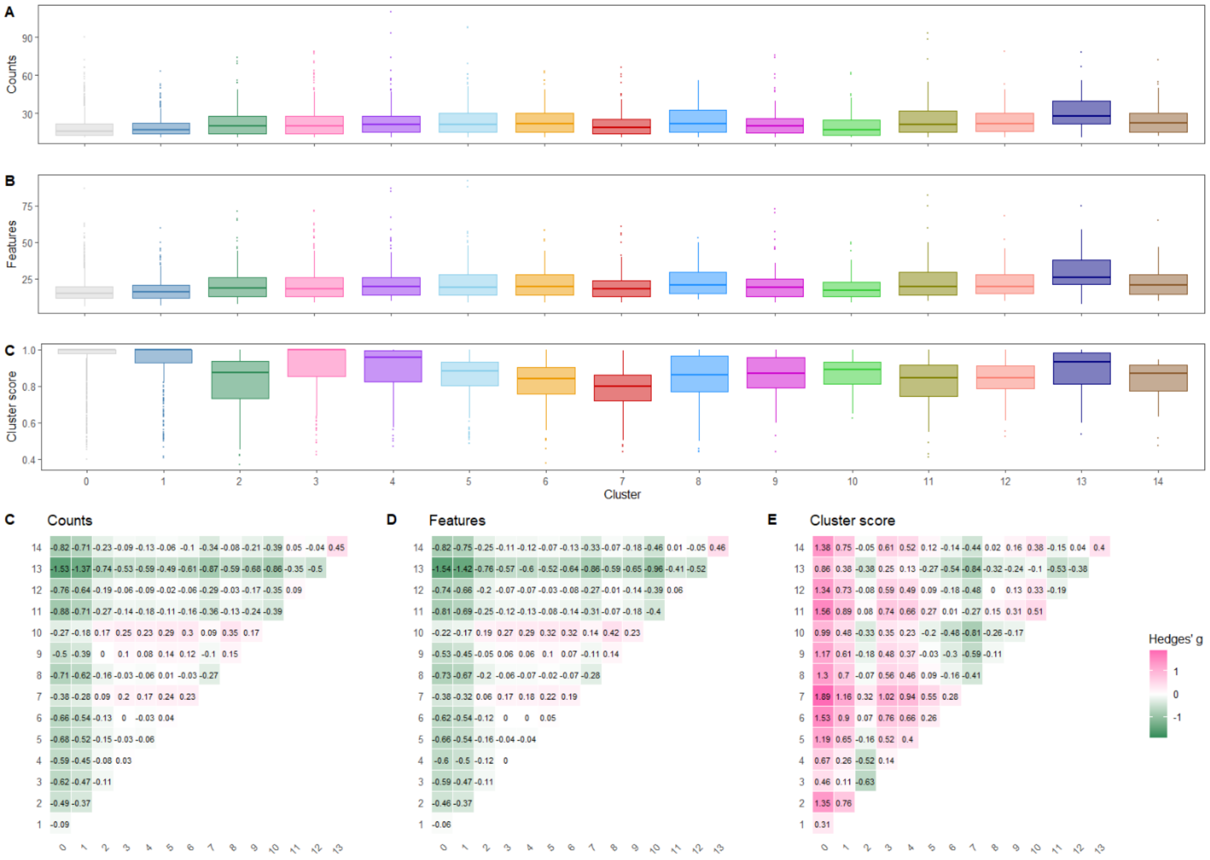

Figure S4: Cluster quality assessment of SSNS dataset. a-c) Box plots of the UMI counts-, features-, and cluster scores, respectively, for the identified clusters. **c-e)** Heatmaps showing the effect size (Hedges' g) of pairwise cluster comparisons for UMI counts-, features-, and cluster scores, respectively. Negative effect size indicate lower column values; positive effect size indicate higher column values.

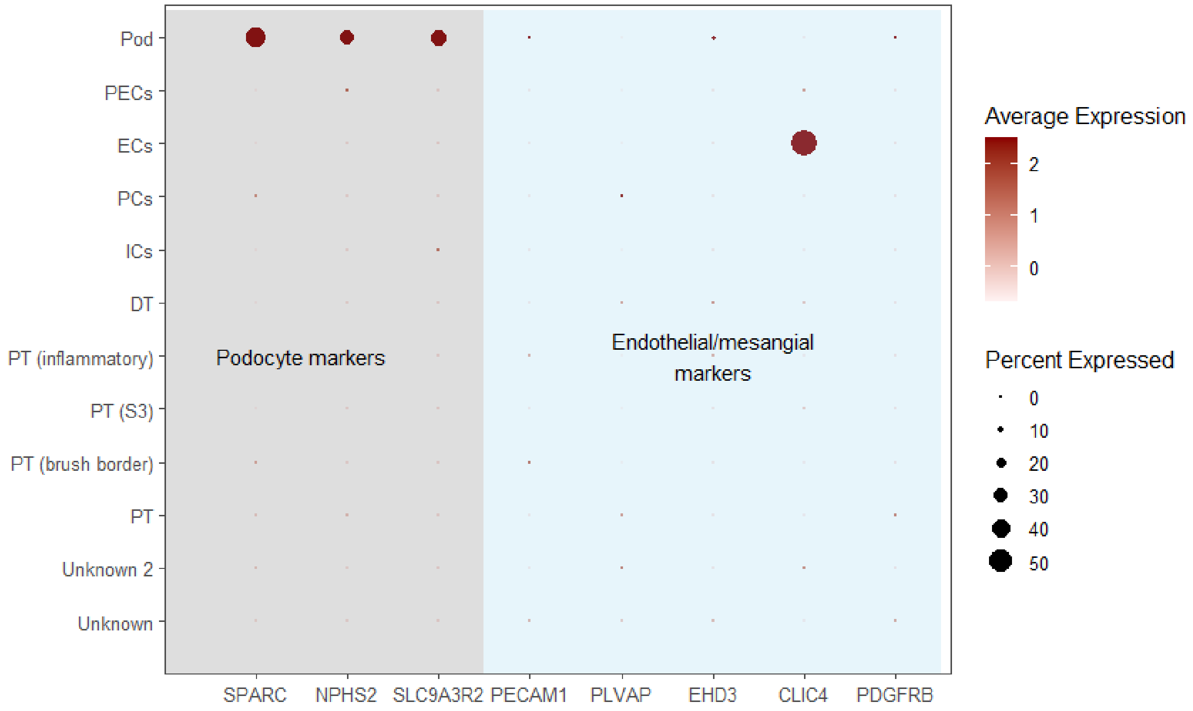

Figure S5: Cluster marker genes for glomerular cells in SSNS dataset. Balloon plot showing the average expression and percentage of cells expressing podocyte markers (gray panel) and endothelial/mesangial markers (blue panel). Podocyte cluster expresses podocyte markers but not endothelial/mesangial cell markers. Abbreviations: PT = proximal tubule, DT = distal tubule, ICs = intercalated cells, PCs = principal cells, ECs = endothelial cells, PECs = parietal epithelial cells, Pod = podocyte.

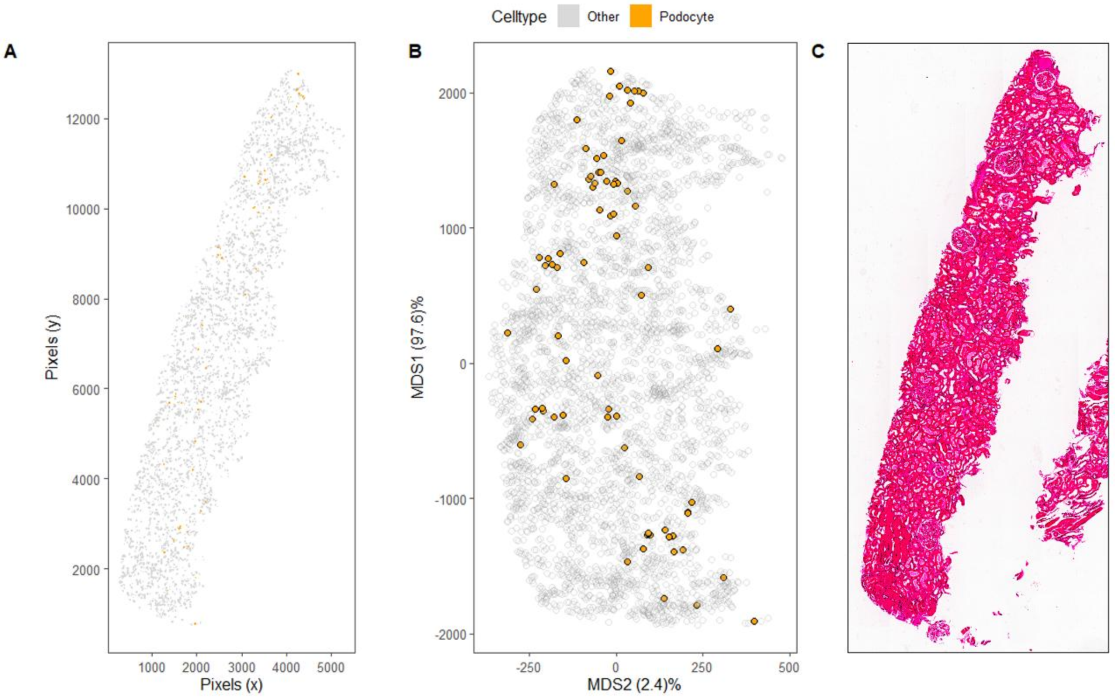

Figure S6: Spatial visualization of podocytes in SSNS patient biopsy. a) Plot of segmented nuclei using raw image coordinates with podocytes highlighted in orange. **b)** Multidimensional scaling (MDS) of nuclei centroid distance matrix (µm) retains spatial information and enhances visual enhancement of podocytes. **c)** H&E stained biopsy. Podocyte locations in (a) and (b) correspond to glomeruli in the biopsy.

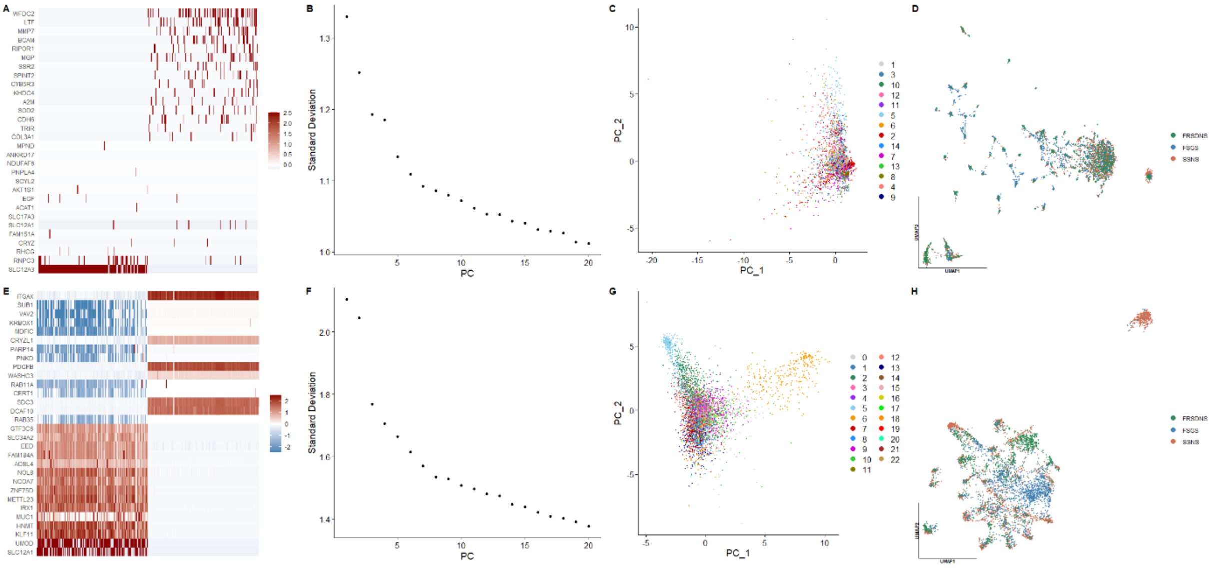

Figure S7: Data integration using CCA. a-d) snRNA-seq clustering of merged, but not integrated, datasets. **e-h)** snRNA-seq clustering of merged and integrated datasets. **a, e)** Heatmaps showing the genes and scaled expression for the top 15 loading scores in the positive and negative direction along the first principal component. **b, f)** Elbow plots showing the reduction in the variation as a function of the number of principal components. **c, g)** PCA plots of the identified clusters along the first two principal components. **d, h)** UMAP embeddings for all three patients.

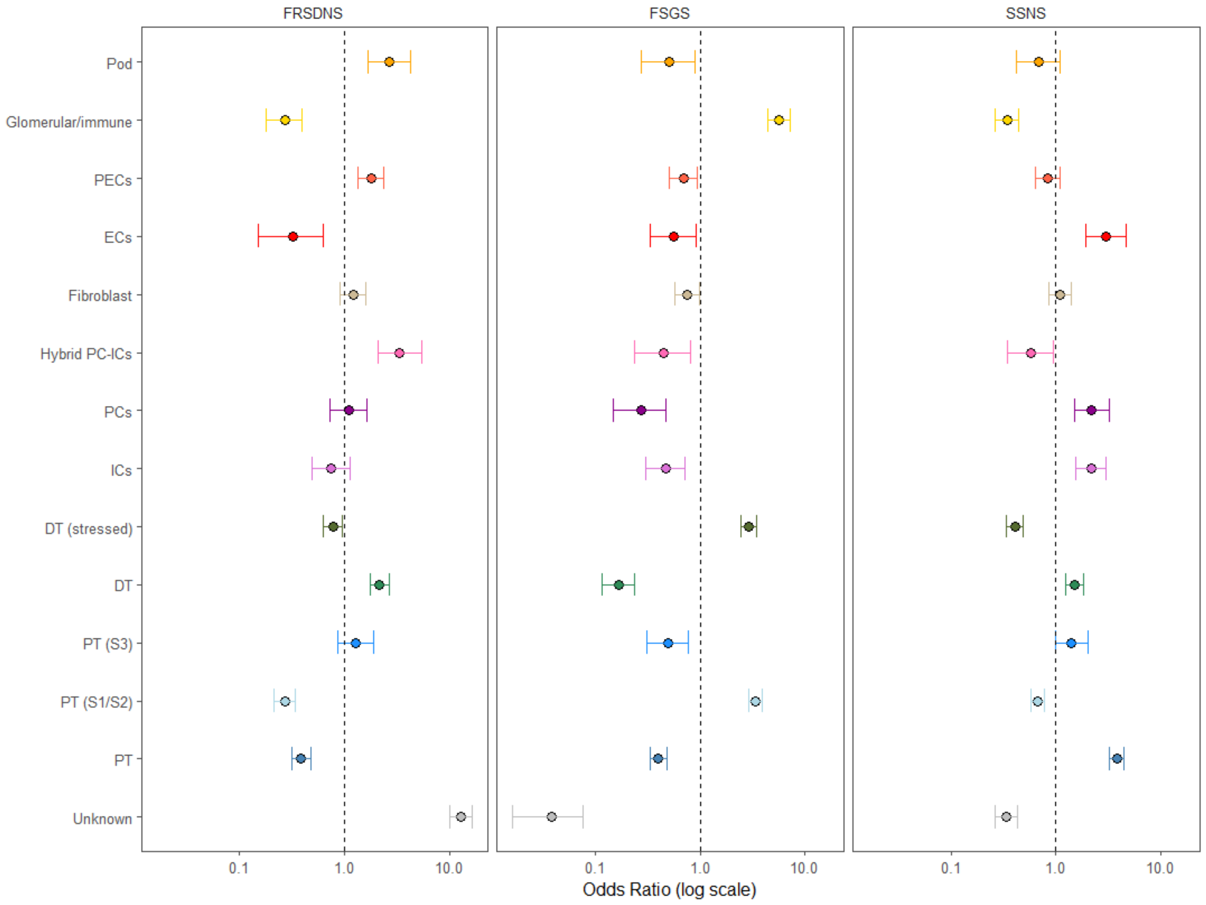

Figure S8: Over/underrepresentation of cell types/states for the three patients. After assigning cell types/states to the clusters in the integrated patient dataset, a Fishers' exact test was carried out to identify cell types/states that were over- or underrepresented in each patient. Dots and error bars indicate odds ratio ± 95 % C.I. Odds ratio < 1 indicates underrepresentation and odds ratio > 1 indicates overrepresentation.

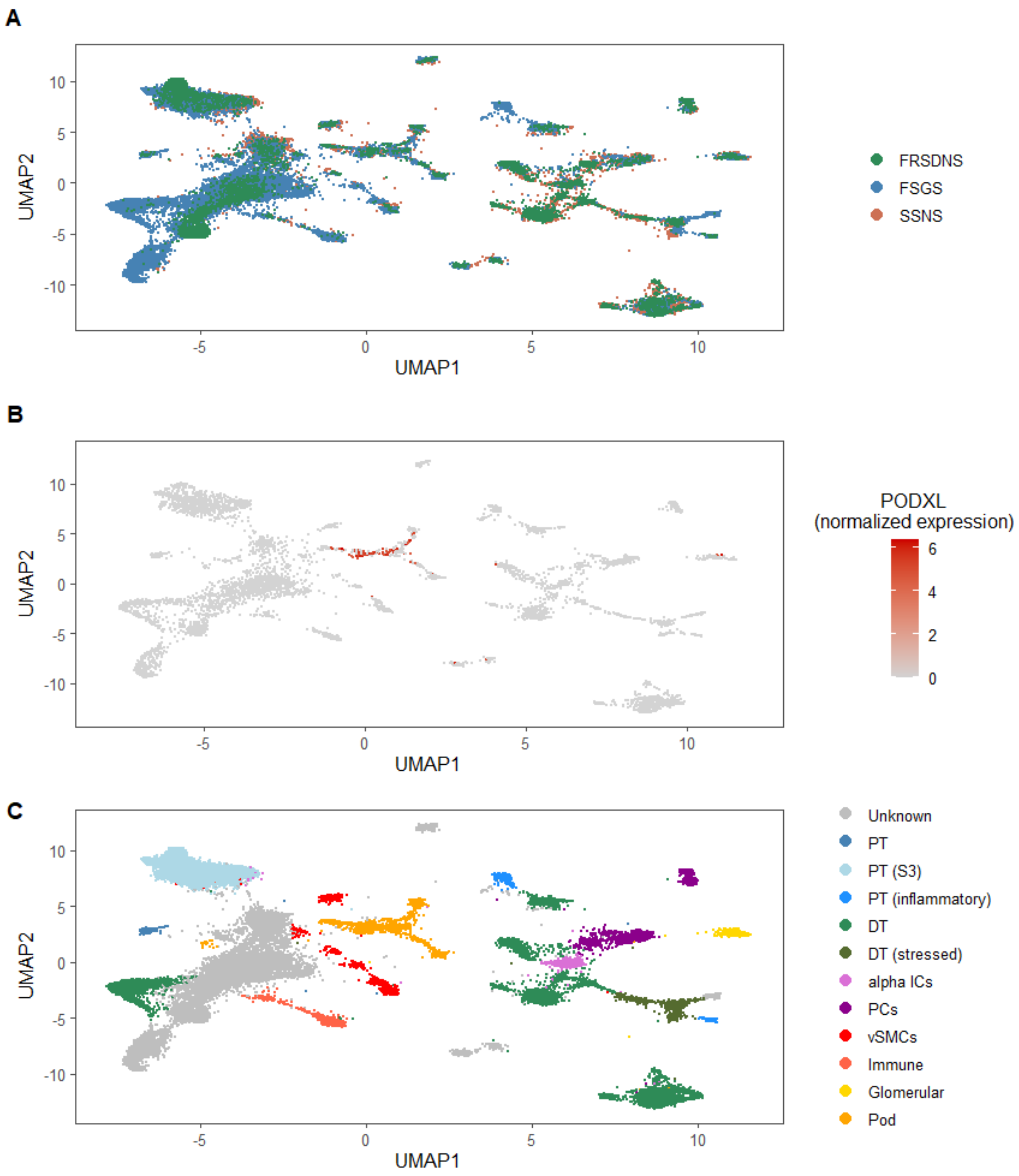

Figure S9: UMAP embeddings of integrated dataset based on 8x8 µm binning. a) UMAP of 8x8 bins showing patient origin. **b)** UMAP of 8x8 bins showing expression of canonical podocyte marker gene *PODXL.* **c)** UMAP showing cell type/state assignment.

Supplementary Text

Discussion

This communication presents a cutting-edge approach for doing spatial transcriptomics on archival kidney biopsies. We reason that this method is obtainable for the wider research community; the analysis was carried out entirely on a 32GB RAM system in an acceptable timeframe (approximately 30 minutes start to finish). The most computationally expensive step will be image segmentation using StarDist. To reduce memory requirements, consider dividing the image into smaller tiles by adjusting the "n_tiles" parameter. Furthermore, users may need to tweak the other parameters in the pre-trained StarDist model for optimal results.

Low data quality is of concern when working with archival FFPE tissue, with gene expression being very sparse and noisy. One manifestation of the low data quality we encountered was that individual patient datasets were sensitive to filtering on counts per nucleus. We recommend others to evaluate the cluster quality from different count thresholds in individual patient datasets before proceeding to integration. Although we were limited by tissue scarcity and did not include DV200 measurements, we recommend others assess the RNA quality of their samples using DV200 prior to sequencing. Additionally, imputation methods specifically designed for single-cell data can be used to regain lost information, but we urge caution. Although not presented here, Markov Affinity-based Graph Imputation of Cells (MAGIC) [S9] has performed well on our datasets.

Supplementary References

S1. 10x Genomics. *Visium HD Spatial Gene Expression Reagent Kits Handbook*. 2024. https://cdn.10xgenomics.com/image/upload/v1734572019/support-documents/CG000684_VisiumHDFFPETissuePrepHandbook_RevB.pdf

S2. 10x Genomics. *Visium HD Spatial Gene Expression Reagent Kits*. 2024. https://cdn.10xgenomics.com/image/upload/v1726587883/support-documents/CG000685_VisiumHD_GeneExpression_UserGuide_RevB_1.pdf

S3. Element Biosciences. *AVITI System User Guide*. 2025. https://go.elementbiosciences.com/aviti-system-user-guide-ma-00008

S4. Virshup I, Bredikhin D, Heumos L, et al. The scverse project provides a computational ecosystem for single-cell omics data analysis. *Nature Biotechnology*. 2023/05/01 2023;41(5):604-606. doi:10.1038/s41587-023-01733-8

S5. 10x Genomics. Segmentation for Visium HD: Analysis Guide. https://www.10xgenomics.com/analysis-guides/segmentation-visium-hd. Accessed 30.05.2025.

S6. Hao Y, Stuart T, Kowalski MH, et al. Dictionary learning for integrative, multimodal and scalable single-cell analysis. *Nature Biotechnology*. 2024/02/01 2024;42(2):293-304. doi:10.1038/s41587-023-01767-y

S7. Andreatta M, Carmona SJ. UCell: robust and scalable single-cell gene signature scoring. Comput Struct Biotechnol J. September 2021;19:3796-3798. doi:10.1016/j.csbj.2021.06.043.

S8. Wickham H, Sievert C. *ggplot2: elegant graphics for data analysis*. vol 10. springer New York; 2009.

S9. van Dijk D, Sharma R, Nainys J, et al. Recovering Gene Interactions from Single-Cell Data Using Data Diffusion. *Cell*. 2018;174(3):716-729.e27. doi:10.1016/j.cell.2018.05.061

S4: STROBE checklist

STROBE Statement—checklist of items that should be included in reports of observational studies

	Item No.	Recommendation	Page No.	Relevant text from manuscript
**Title and abstract**	1	(*a*) Indicate the study's design with a commonly used term in the title or the abstract	1	See title
		(*b*) Provide in the abstract an informative and balanced summary of what was done and what was found	NA	
**Introduction**				
Background/rationale	2	Explain the scientific background and rationale for the investigation being reported	1	See first paragraph
Objectives	3	State specific objectives, including any prespecified hypotheses	1	See second paragraph
**Methods**				
Study design	4	Present key elements of study design early in the paper	1	
Setting	5	Describe the setting, locations, and relevant dates, including periods of recruitment, exposure, follow-up, and data collection	NA	
Participants	6	(*a*) *Cohort study*—Give the eligibility criteria, and the sources and methods of selection of participants. Describe methods of follow-up	NA	
		Case-control study—Give the eligibility criteria, and the sources and methods of case ascertainment and control selection. Give the rationale for the choice of cases and controls	NA	
		Cross-sectional study—Give the eligibility criteria, and the sources and methods of selection of participants	NA	
		(*b*) *Cohort study*—For matched studies, give matching criteria and number of exposed and unexposed	NA	

		Case-control study—For matched studies, give matching criteria and the number of controls per case	NA
Variables	7	Clearly define all outcomes, exposures, predictors, potential confounders, and effect modifiers. Give diagnostic criteria, if applicable	NA
Data sources/ measurement	8*	For each variable of interest, give sources of data and details of methods of assessment (measurement). Describe comparability of assessment methods if there is more than one group	NA
Bias	9	Describe any efforts to address potential sources of bias	NA
Study size	10	Explain how the study size was arrived at	NA

Continued on next page

Quantitative variables	11	Explain how quantitative variables were handled in the analyses. If applicable, describe which groupings were chosen and why	NA	
Statistical methods	12	(*a*) Describe all statistical methods, including those used to control for confounding	NA	
		(*b*) Describe any methods used to examine subgroups and interactions	NA	
		(*c*) Explain how missing data were addressed	NA	
		(*d*) *Cohort study*—If applicable, explain how loss to follow-up was addressed<br>*Case-control study*—If applicable, explain how matching of cases and controls was addressed<br>*Cross-sectional study*—If applicable, describe analytical methods taking account of sampling strategy	NA	
		(*e*) Describe any sensitivity analyses	NA	
**Results**				
Participants	13*	(a) Report numbers of individuals at each stage of study—eg numbers potentially eligible, examined for eligibility, confirmed eligible, included in the study, completing follow-up, and analysed	1	N = 3
		(b) Give reasons for non-participation at each stage	NA	
		(c) Consider use of a flow diagram	NA	
Descriptive data	14*	(a) Give characteristics of study participants (eg demographic, clinical, social) and information on exposures and potential confounders	1	See supplementary material S2
		(b) Indicate number of participants with missing data for each variable of interest	NA	
		(c) *Cohort study*—Summarise follow-up time (eg, average and total amount)	NA	
Outcome data	15*	*Cohort study*—Report numbers of outcome events or summary measures over time	*NA*	
		Case-control study—Report numbers in each exposure category, or summary measures of exposure	*NA*	
		Cross-sectional study—Report numbers of outcome events or summary measures	*NA*	

Main results	16	(*a*) Give unadjusted estimates and, if applicable, confounder-adjusted estimates and their precision (eg, 95% confidence interval). Make clear which confounders were adjusted for and why they were included	NA
		(*b*) Report category boundaries when continuous variables were categorized	NA
		(*c*) If relevant, consider translating estimates of relative risk into absolute risk for a meaningful time period	NA

Continued on next page

Other analyses	17	Report other analyses done—eg analyses of subgroups and interactions, and sensitivity analyses	2	See paragraphs 2-4
**Discussion**				
Key results	18	Summarise key results with reference to study objectives	2	See first paragraph
Limitations	19	Discuss limitations of the study, taking into account sources of potential bias or imprecision. Discuss both direction and magnitude of any potential bias	2, 3	See paragraph six
Interpretation	20	Give a cautious overall interpretation of results considering objectives, limitations, multiplicity of analyses, results from similar studies, and other relevant evidence	3	See paragraph seven
Generalisability	21	Discuss the generalisability (external validity) of the study results	2	Because of the low sample size, it is important to emphasize that this analysis only serves as proof-of-concept for the detection of intra-cell type differential expression across patients. As such, we do not relate these differences back to the respective clinical courses.
**Other information**				
Funding	22	Give the source of funding and the role of the funders for the present study and, if applicable, for the original study on which the present article is based	4	Research council of Norway (334097 to HPM).

*Give information separately for cases and controls in case-control studies and, if applicable, for exposed and unexposed groups in cohort and cross-sectional studies.

Note: An Explanation and Elaboration article discusses each checklist item and gives methodological background and published examples of transparent reporting. The STROBE checklist is best used in conjunction with this article (freely available on the Web sites of PLoS Medicine at http://www.plosmedicine.org/, Annals of Internal Medicine at http://www.annals.org/, and Epidemiology at http://www.epidem.com/). Information on the STROBE Initiative is available at www.strobe-statement.org
