# Supplementary material for: Reconstruction of Single-Cell Spatial Transcriptomes in Archival Kidney Biopsies
Source: Kidney Int Rep. 2025 Dec 5;11(2):103713. doi: 10.1016/j.ekir.2025.11.036 (PMC12805023; doi:10.1016/j.ekir.2025.11.036)
Supplement: Supplementary File (PDF) — Supplementary Methods. Supplementary References. Supplementary Text. Figure S1. snRNA-seq processing of SSNS dataset. Figure S2. snRNA-seq processing of FSGS dataset. Figure S3. snRNA-seq processing of FRSDNS dataset. Figure S4. Cluster quality assessment of SSNS dataset. Figure S5. Cluster marker genes for glomerular cells in SSNS dataset. Figure S6. Spatial visualization of podocytes in SSNS patient biopsy. Figure S7. Data integration using CCA. Figure S8. Over/underrepresentation of cell types/states for the 3 patients. Figure S9. UMAP embeddings of integrated dataset based on 8 x 8 μm binning. Table S1. Parameters used for unsupervised clustering. Table S2. Patients’ clinical characteristics. STROBE checklist. [file mmc2.pdf]

Supplementary Methods

Briefly, FFPE biopsies from three INS patients - one each with steroid-sensitive (SSNS), frequent-relapsing/steroid-dependent (FRSDNS), and focal segmental glomerulosclerosis (FSGS) - were sequenced using Visium HD. For each patient, 4 μm^2 capture areas were custom-binned to StarDist-segmented nuclei, and the resulting single-nucleus transcriptomes were clustered into distinct cell types. Datasets were then merged to identify conserved cell types across patients, and nuclei-based binning was compared with conventional 64 μm^2 binning.
